# An aberrant medial basal segmental pulmonary artery (A7b) behind the superior segmental pulmonary vein (V6) in a patient undergoing right superior segment (S6) segmentectomy

**DOI:** 10.1093/jscr/rjab294

**Published:** 2021-07-14

**Authors:** Yoshiaki Takase, Hiroyoshi Tsubochi, Ei Yamaki, Osamu Kawashima

**Affiliations:** Department of Thoracic Surgery, Sapporo Medical University School of Medicine, South-1, West-16, Chuo-ku, Sapporo 060-8543, Japan; Department of Thoracic Surgery, Shibukawa Medical Center, 383 Shiroi, Shibukawa Gunma, 377-0280, Japan; Department of Thoracic Surgery, Jichi Medical University, 3311-1 Yakushiji, Shimotsuke, Tochigi 329-0498, Japan; Department of Thoracic Surgery, Shibukawa Medical Center, 383 Shiroi, Shibukawa Gunma, 377-0280, Japan; Department of Thoracic Surgery, Shibukawa Medical Center, 383 Shiroi, Shibukawa Gunma, 377-0280, Japan

## Abstract

Herein, we report the first case of a patient with lung cancer with an aberrant medial basal segmental pulmonary artery (A7b) behind the superior segmental pulmonary vein (V6) who underwent right superior segment (S6) segmentectomy via uniportal video-assisted thoracoscopic surgery (uVATS). A 56-year-old man with a right lower lobe pure ground-glass nodule (GGN), measuring 12 mm in diameter on computed tomography (CT) had an aberrant A7b branching from the basal pulmonary artery, which was located behind the V6 as detected on 3D CT. The right S6 segmentectomy, via uVATS, for the GGN was performed. The postoperative course was uneventful. The final pathological diagnosis was invasive adenocarcinoma (p-T1bN0M0, stage IA2) with no evidence of disease recurrence at 3-month follow-up. Thoracic surgeons should be aware of the possibility of damaging the A7b when dividing the V6 for S6 segmentectomy, especially during uVATS because of insufficient dorsal visibility.

## INTRODUCTION

Very few published reports have documented lung resection with an anatomical abnormality of the medial basal segmental pulmonary artery (A7) [1–3]. To the best of our knowledge, there are no reports that detail the discovery of an isolated anatomical abnormality of the A7b located behind the superior segmental pulmonary vein (V6). Attention should be paid to vessel anomalies, particularly during dissection of the dorsal side of the hilum via uniportal video-assisted thoracoscopic surgery (uVATS), because the dorsal field of view during uVATS tends to be insufficient compared with that during multiportal VATS (mVATS). Herein, we report a rare case of an aberrant A7b that was detected behind the V6 in a patient who underwent right superior segment (S6) segmentectomy via uVATS for lung cancer.

**Figure 1:**
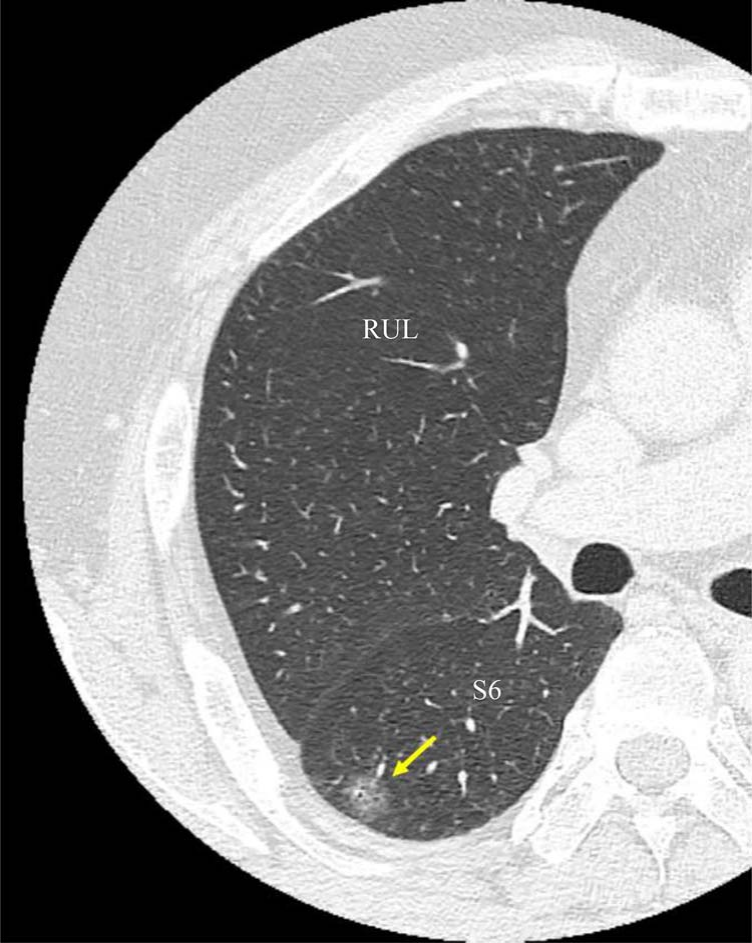
An image from CT scan taken in the axial plane. The tumor is located in the S6 of the right lung (arrow). RUL, right upper lobe; S6, superior segment.

**Figure 2:**
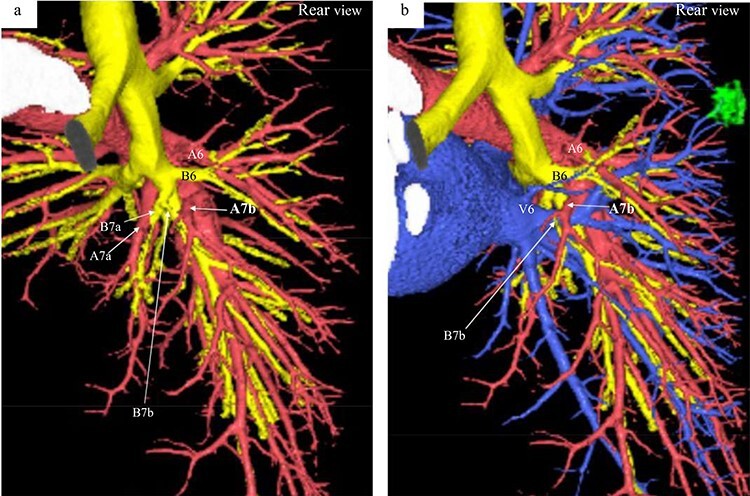
Three-dimensional CT angiography and bronchography images. (**a**) Rear-view CT angiography image of the PA and bronchi. (**b**) Rear-view CT image of the PA, PV, and bronchi. Reconstructed images of the pulmonary arteries (red), pulmonary veins (blue), bronchi (yellow) and tumor (green). A, pulmonary artery; B, bronchus; V, pulmonaryvein.

## CASE PRESENTATION

A 56-year-old man was referred to our department for a right lower ground-glass nodule (GGN). Computed tomography (CT) revealed a pure GGN measuring 12 mm in diameter in the S6 of the right lung (Fig. 1). A pathological diagnosis could not be determined via transbronchial tumor biopsy. Three months after the initial investigation, CT revealed that the GGN had not shrunk, and a highly differentiated adenocarcinoma was strongly suspected. No hilar or mediastinal lymph nodes, or other organ metastases were observed on positron emission tomography/CT, or contrast-enhanced magnetic resonance imaging of the brain. Preoperative 3D CT revealed an aberrant A7b branching from the basal pulmonary artery (PA) and running behind the V6 (Fig. 2). The A7a and A7b subsegmental pulmonary arteries were located on the ventral and dorsal sides of the basal vein, respectively. Because lung cancer was highly suspected, surgery was scheduled.

**Figure 3:**
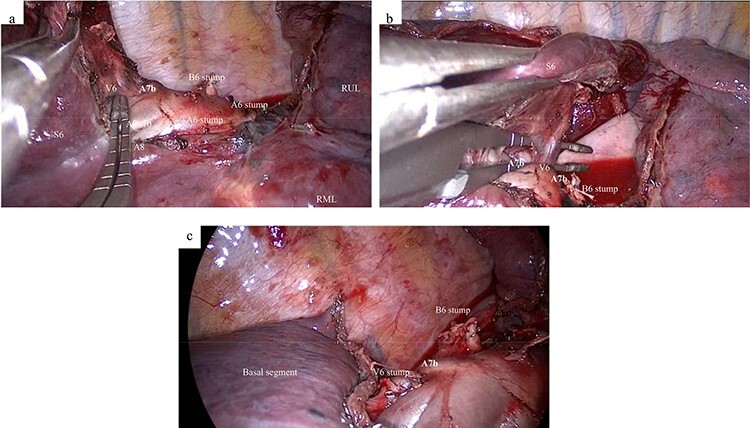
Intraoperative findings. (**a**) After division of the B6 and A6, an aberrant A7b is detected on the cranial side of the V6. (**b**) After inserting the dissecting forceps between the V6 and the aberrant A7b, the A7b is detected behind the V6. (**c**) After dissection of the V6 and lung parenchyma between the S6 and basal segment. A6, superior segmental pulmonary artery; A7, medial basal segmental pulmonary artery; B6, superior segmental bronchus; RML, right middle lobe; RUL, right upper lobe; S6, superior segment; V6, superior segmental pulmonaryvein.

Under general anesthesia, the patient was placed in a left lateral decubitus position and underwent single lung ventilation via a double lumen tube. A 4-cm incision was made along the anterior axillary line at the fifth intercostal space. At first, wedge resection of the S6 was performed, and an intraoperative frozen section pathological analysis revealed that the tumor was compatible with a lung adenocarcinoma. Therefore, we decided to perform S6 segmentectomy. After dividing the fissure between the S6 and posterior segment (S2) in the upper lobe, the A6 was divided. After the hilar lymph node (#12L) and the segmental lymph node (#13) around the B6 was dissected, the B6 was divided with a stapling device (Fig. 3a). The aberrant A7b was observed behind the V6 (Fig. 3b). After the caudal side of the A7b was exfoliated, the V6 was completely exposed (Fig. 3c). The V6 was divided with a stapling device without damaging the aberrant A7b. The intersegmental plane was dissected using the stapling device along the inflation and deflation lines. In addition, the interlobar lymph nodes (#11s) were dissected and the upper lobar lymph nodes (#12u) were sampled. The total operation time was 187 minutes, and the total blood loss volume was minimal. The postoperative course was uneventful. The final pathological diagnosis was invasive adenocarcinoma (p-T1bN0M0, stage IA2). At the 3-month postoperative follow-up, there was no evidence of disease recurrence.

## DISCUSSION

Only three case reports have detailed an abnormal A7 branching in patients who underwent lung resection [1–3]. One report showed an aberrant mediastinal inferior lobar branch [A6 + the common basal artery (A7–10)] originating from the right main PA [1]. The remaining two reports also revealed A7 branching from the right main PA in patients who underwent lung resection for lung cancer [2, 3]. In these cases, the A7 and A7a branched from the main PA and the A7b branched as usual from the A8 + 9 + 10. The present case is unique because only the A7b branched from the A8 + 9 + 10 and was located behind theV6.

According to a previous report, the branching pattern of the B7 and A7 can be classified into four types: B^7^a, B^7^ab, B^7^b, and BX^7^. The B^7^a type is more common (74.8%) than the B^7^ab (14.8%), B^7^b (4.8%) and BX^7^ types (5.6%) [4]. Of the least common A7b pattern type (4.8%), only seven cases (2.6%) had their A7b diverged from the cranial side of the V6, as in our case. In the present case, the PA pattern and bronchus branching were compatible with the B^7^ab type, in which there are two bronchi (B7a and B7b) and two arteries (A7a and A7b). To the best of our knowledge, no previous reports have detailed a B^7^ab type, where the A7b branches behind theV6.

The uVATS is a less invasive procedure with minimal postoperative pain compared with mVATS; however, more technical skills are required to perform lobectomies and segmentectomies via uVATS than via mVATS [5]. One disadvantage of uVATS using the anterior approach is poor visibility of the dorsal side of the lung compared with mVATS [6]. Therefore, when aberrant PAs are located on the dorsal side of the lung, the risk of injury to such a PA might be higher during uVATS than during mVATS. When exfoliating the V6 using the anterior approach, careless manipulation can cause injury to an aberrant A7b. If 3DCT cannot be performed preoperatively because of contrast medium allergy or renal dysfunction, it may be appropriate to make the single incision slightly to the dorsal side in order to improve the dorsal field of view. Thoracic surgeons should consider the possibility of an aberrant A7b during anatomical segmentectomy of the S6 in the right lowerlobe.

In conclusion, this is the first report detailing an aberrant A7b located behind the V6, detected prior to uVATS, in a patient with lung cancer. Thoracic surgeons should be aware of the possibility of damaging the A7b when dividing the V6 for S6 segmentectomy, especially during uVATS because of insufficient dorsal visibility. Preoperative 3DCT is effective at obtaining accurate information on anatomical variations in the pulmonary vessels, aiding safe lung surgery.
